# Supplementary material for: Characterization of MYBL1 Gene in Triple-Negative Breast Cancers and the Genes’ Relationship to Alterations Identified at the Chromosome 8q Loci
Source: Int J Mol Sci. 2024 Feb 22;25(5):2539. doi: 10.3390/ijms25052539 (PMC10932083; doi:10.3390/ijms25052539)

Supplemental Figure S3: DOMAINS associated with MYBL1 (Myb-related protein A). Carboxyl-terminal region demonstrates the regulatory region with designation of phosphorylation, acetylation and ubiquitylation sites (rectangular area) [23].

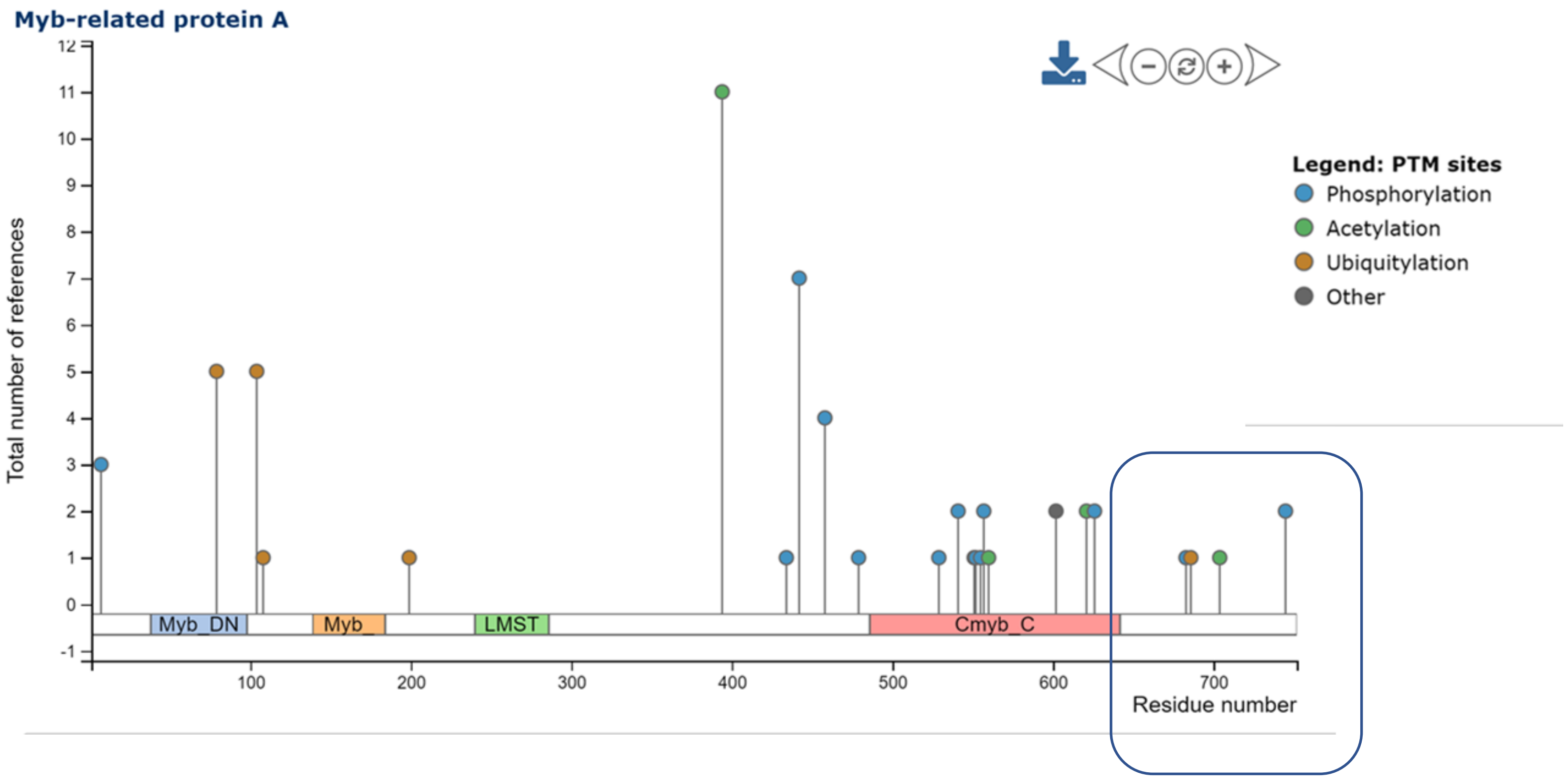

Supplement: Supplementary file 1 [file ijms-25-02539-s001.zip › Supplemental Figure S3.pdf]
