# Supplementary material for: Characterization of MYBL1 Gene in Triple-Negative Breast Cancers and the Genes’ Relationship to Alterations Identified at the Chromosome 8q Loci
Source: Int J Mol Sci. 2024 Feb 22;25(5):2539. doi: 10.3390/ijms25052539 (PMC10932083; doi:10.3390/ijms25052539)
Supplement: Supplementary file 1 [file ijms-25-02539-s001.zip › Supplemental Figures S1 and S2.pdf]

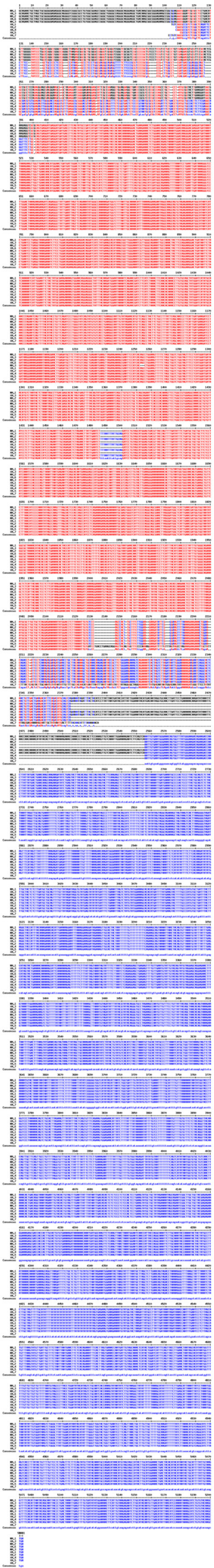

**Supplemental Figure S1-** MultalinTM Sequence alignment comparison of all Refence Sequence transcript variants

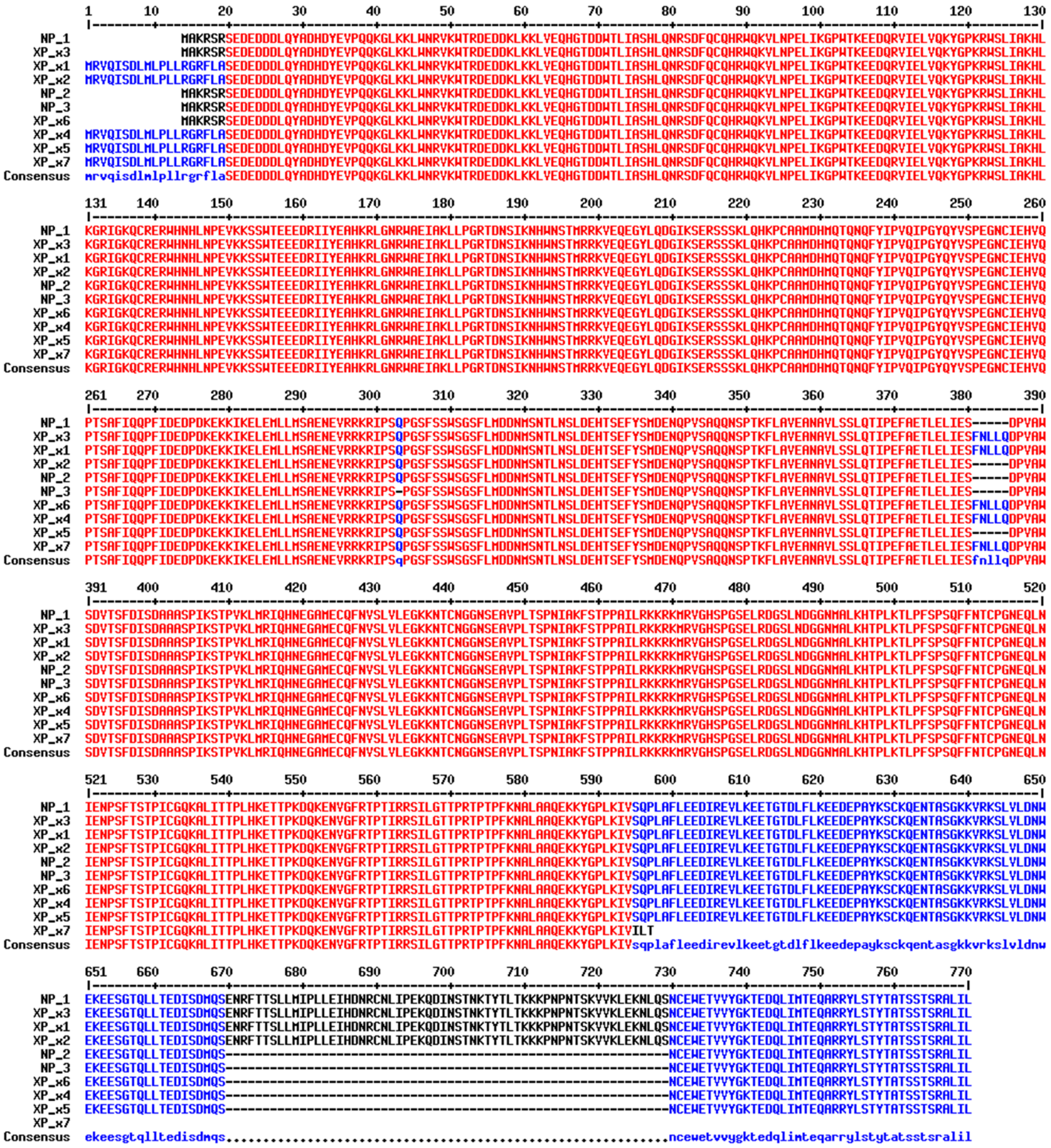

**Supplemental Figure S2-** MultalinTM Sequence alignment comparison of all Refence Sequence protein isoforms
