# Supplementary material for: Characterization of MYBL1 Gene in Triple-Negative Breast Cancers and the Genes’ Relationship to Alterations Identified at the Chromosome 8q Loci
Source: Int J Mol Sci. 2024 Feb 22;25(5):2539. doi: 10.3390/ijms25052539 (PMC10932083; doi:10.3390/ijms25052539)
Supplement: Supplementary file 1 [file ijms-25-02539-s001.zip › Supplemental Table S1.pdf]

Supplemental Table S1: Affymetrix microarray results for genes on chromosome 8q affected by MYBL1 knockdown in MDA MB231 cells. Highlighted genes validate via PCR and Western analyses. Fold change is the difference in transcript levels (obtained from microarray); control values vs knockdown [16].

GENE	No knock down (RNA levels for the gene) LOG 2 VALUES	mybl1 knock down (RNA levels for the gene LOG2 VALUES	FOLD CHANGE	CHROMOSOME
**MYBL1**	12.2	10.2	4X down	8q13.1
RMDN1 2	10.87	12.69	6.8X up	8q21.3
MRPL13	10.12	12.89	6.8X up	8q24.12
MAF-1	12.12	10.21	3.7X dw	8q24.3
SDC2	12.61	10.9	3X down	8q22.1
GGH 1	10.1	12.5	5.2X up	8q12.3
**MYC**	12.7	10.6	4.4X down	8q24.21
**BOP1**	11.3	8.5	7X down	8q24.3
PUF60	10	8.3	3X down	8q24.3
SNX16 3	9.66	11.4	3.3X up	8q21.13
IMPAD1/BPNT2 4	11.44	9.3	4X down	8q12.1
AZIN1	15.2	12.9	4.8X down	8q22.3
EBAG9	12.4	14.4	3.9X up	8q23.2
MRPS28	10.3	12.1	3.X up	8q21.13
**VCPIP1**	10.1	9	2.1X down	8q13.1
